# Identification and Characterization of Edible Cricket Peptides on Hypertensive and Glycemic In Vitro Inhibition and Their Anti-Inflammatory Activity on RAW 264.7 Macrophage Cells

**DOI:** 10.3390/nu12113588

**Published:** 2020-11-23

**Authors:** Felicia Hall, Lavanya Reddivari, Andrea M. Liceaga

**Affiliations:** 1 Protein Chemistry and Bioactive Peptides Laboratory, Purdue University, West Lafayette, IN 47907, USA; hall351@purdue.edu; 2 Department of Food Science, Purdue University, 745 Agriculture Drive, West Lafayette, IN 47907, USA; lreddiva@purdue.edu

**Keywords:** cricket protein, cationic peptides, ACE inhibition

## Abstract

Recent studies continue to demonstrate the potential of edible insects as a protein base to obtain bioactive peptides applicable for functional food development. This study aimed at identifying antihypertensive, anti-glycemic, and anti-inflammatory peptides derived from the in vitro gastrointestinal digests of cricket protein hydrolysates. After sequential fractionation, the protein digest subfraction containing the lowest molecular weight (<0.5 kDa), hydrophobic (C18) and cationic peptides (IEX) was found responsible for the most bioactivity. The cationic peptide fraction significantly reduced (*p* < 0.05) α-amylase, α-glucosidase, and angiotensin converting enzyme (ACE) activity in vitro, and also inhibited the expression of NF-κB in RAW 264.7 macrophage cells. A total of 28 peptides were identified with mass spectrometry (LC–MS/MS) and de novo sequencing from the potent fraction. Three novel peptides YKPRP, PHGAP, and VGPPQ were chosen for the molecular docking studies. PHGAP and VGPPQ exhibited a higher degree of non-covalent interactions with the enzyme active site residues and binding energies comparable to captopril. Results from this study demonstrate the bioactive potential of edible cricket peptides, especially as ACE inhibitors.

## 1. Introduction

Cardiovascular diseases (CVD) are currently the number one cause of death globally, estimating about 18 million lives per year. Diets high in saturated fats and sugar are a well-established risk factor in coronary heart disease, since these pro-inflammatory diets are linked to other significant risk factors such as obesity, hypertension, and diabetes [1]. Hence, anti-inflammatory diets are recommended to prevent, treat, and manage CVD and its related risk factors [1]. While fruits and vegetables are undoubtedly associated with preventing CVD risk factors, food proteins and peptides with biological activities were also identified, suggesting their potential use as nutraceuticals and functional food ingredients for health promotion [2].

In recent years, consumption of edible insects has increased in western cultures, as a response to demand for alternative, more sustainable, and eco-friendly resources. The application of insect protein in food formulations is widely reported in the literature [3,4,5]. However, there is an emerging interest in the potential of insect proteins as sources of bioactive agents. Studies are continuously demonstrating various biologically active properties such as antidiabetic, antihypertensive, antioxidant, and anti-inflammatory properties [6,7,8]. Typically, the insect-derived protein components are in the form of extracts (i.e., water extracts, protein hydrolysates, or buffer extracts) and in other cases isolated/purified peptide or peptide mixtures. Various cricket protein preparations exhibited multiple bioactive properties in vitro, yet enzymatic hydrolysis stands as one of the more effective methods used to liberate active peptides [9]. We speculate that the active polypeptide fractions of insects are more readily available after proteolysis, as commonly seen with other protein-derived biopeptides [10,11]. The protein hydrolysate is typically free of the insect body (i.e., exoskeleton), which contains high amounts of proteins but are complexed with the chitin polymerized matrix. For example, Vercruysse et al. [12] reported that enzymatic hydrolysis was necessary to release bioactive peptides with ACE inhibitory activity from cotton leafworm (*Spodoptera littoralis*). Gastrointestinal digestion of the cotton leafworm protein hydrolysates, using pepsin, trypsin, and chymotrypsin, improved the IC_50_ values significantly from 73 mg/mL to 0.7 mg/mL, representing high activity. The potency of the ACE inhibitory peptides (Ala-Val-Phe) from the protein hydrolysate was confirmed to have antihypertensive activity in vivo [13].

In our own work, we confirmed that chitin was successfully removed when developing tropical banded cricket protein hydrolysates. In addition, these hydrolysates demonstrated better in vitro antioxidant, ACE, and DPP-IV inhibiting activity, compared to the non-hydrolyzed cricket protein; the activity increased further after simulated gastrointestinal digestion [7]. Increased hydrolysis time showed the best bioactivity overall, owing to the smaller molecular weight peptides. The present study aimed to identify and characterize the bioactive peptides in cricket protein, which responsible for the ACE and DPP-IV-inhibiting activity, while we also evaluate their anti-inflammatory activity on RAW 264.7 macrophage cells. Finally, we used in silico analysis to help elucidate the structural interactions between these bioactive peptides and ACE.

## 2. Materials and Methods

### 2.1. Materials

Unless specified, all chemical reagents were of analytical grade and obtained from suppliers: MilleporeSigma (St. Louis, MO, USA) and ThermoFisher Scientific (Waltham, MA, USA). Alcalase^®^ (*Bacillus licheniformis*, 2.4 U/g), The Renin Inhibitor Screening Assay Kit, Human Dipeptidyl Peptidase IV (DPP-IV, ≥4500 units/µg protein), and substrate Gly-Pro p-nitroanilide hydrochloride, Angiotensin Converting Enzyme (ACE) from rabbit lung and substrate Hippuryl-L-Histidyl-L-Leucine (HHL), α-Amylase (Type VI-B, ≥5 units/mg), and Rat intestinal acetone powder (α-Glucosidase) were all purchased from MilleporeSigma (St. Louis, MO, USA). Whole, frozen, unpasteurized, adult (6 weeks old) tropical banded crickets (*Gryllodes sigillatus*) were purchased from 3 Cricketeers, LLC (Hopkins, MN, USA). RAW 264.7 macrophage cells were obtained from Novus Biologicals (Littleton, CO, USA), whereas Renilla Luciferase Activity Bioluminescence Detection Kit was purchased from the Promega Corporation (Madison, WI, USA).

### 2.2. Cricket Protein Hydrolysate Preparation

Cricket protein hydrolysates (CPH) and its digests were produced, as previously reported [7]. In brief, crickets were hydrolyzed with alcalase (3.0% E/S), under controlled convection heating for 80 min at 55 °C, followed by heating for 15 min at 95 °C, to terminate proteolysis. After centrifugation, the supernatants (30 mg/mL) were subject to a simulated gastrointestinal digestion (SGID), as reported by You et al. [14], with modifications [15]. SGID was initiated with the gastric phase with pepsin (4% *w*/*w* of protein), then in the intestinal digestion phase with bile salts (10 mg/mL) and pancreatin (4% *w*/*w* protein). Both phases were incubated at the enzyme pH optima, in a water bath, with continuous shaking. Supernatants after filtration were collected, referred to as CPH-digests (CPHD), freeze dried, and stored at −20 °C, until further use.

### 2.3. Molecular Weight Distribution of CPH and CPHD

The molecular weight distributions of CPH before and after in vitro digestion (CPHD) were evaluated following a described protocol [15]. Peptides were separated with Superdex™ Peptides 10/300 GL column (GE Healthcare Bio-Science AB, Uppsala, Sweden) on a Waters e2695 HPLC system, equipped with a PDA detector (Waters Co., Milford, MA, USA). The fractionation ranged from 1000–7000 kDa. In brief, samples (5 mg/mL) were filtered (0.45 µm) before injection (50 µL), at a flow rate of 0.5 mL/min with 100 mM sodium phosphate buffer (pH 7.0). Molecular weight standards were used for molecular weight calculation and ran with the same conditions, including bovine serum albumin (66,400 Da), aprotinin (6511 Da), vitamin B12 (1355 Da), cytidine (243 Da), dl-dithiotheritol (154 Da), and glycine (75 Da). Plots of retention time for molecular weight standards were used to construct the calibration curve, from which molecular distributions were computed. The logarithm of molecular weight (lg MW) and the retention time (Rt) were in a linear relationship and the formula was calculated as Rt = −0.2094 (lg MW) + 1.2747 (R2 = 0.9867, *p* < 0.05).

### 2.4. CPHD Peptide Fractionation

#### 2.4.1. Size Exclusion Chromatography (SEC)

The gastrointestinal digest was re-dissolved in 10 mg/mL elution buffer (20 mM sodium phosphate buffer with 300 mM NaCl, pH 7.3) and filtered (0.45 µm), before being applied to a gel filtration Superdex^®^ Peptide 10/300 GL (30 cm × 10 mm, 13 μm, GE Healthcare Life Sciences, Chicago, IL, USA). Elution was carried out at room temperature on a Waters 2690 HPLC system (Waters Corporation, Milford, MA, USA), equipped with an automatic sample injector and 2998 UV photodiode array (PDA) detector, at a flow rate of 0.5 mL/min. The absorbance of each fraction was measured at a wavelength of 214 nm. After collection, fractions were concentrated, reconstituted in assay buffers, then the bioactivities were measured, as described above.

#### 2.4.2. Reverse-Phased–High-Performance Liquid Chromatography (RP–HPLC)

The fraction with the strongest overall in vitro bioactivity after SEC separation was further purified on an XBridge™ BEH130 C18 column (10 μm, 10 × 150 mm, Waters Inc., Milford, MA, USA), at a flow rate of 1 mL/min. Eluent A: Water/0.1% TFA; eluent B: Acetonitrile/0.1% TFA. After 50 µL of the sample (10 mg/mL) was injected into the C18 column, concentrations of eluent B were increasing as: 0–40 min, 0–100% (*v*/*v*); and 40–50 min, 100% (*v*/*v*). Eluents were monitored at 218 nm, fractions were collected (F1 to F6) and concentrated, and bioactivity was measured, as described above.

#### 2.4.3. Ion Exchange Chromatography (IEX)

The most potent fraction (F6) from RP–HPLC was loaded onto a HiTrap SP HP cation exchange chromatography column (34 µm; GE Healthcare Life Sciences, Marlborough, MA, USA) with a flow rate of 1 mL/min. The sample was sequentially eluted with Citrate Buffer (pH 4.3) with no or 1 M NaCl, and both anion and cationic fractions were collected. Fractions were desalted and concentrated using Pierce™ C18 Tips (100 µL), following the manufactures’ protocol (ThermoFisher Scientific, Walthman, MA, USA). The most potent fraction for the in vitro bioactivities, described above, was assessed for protein sequencing.

### 2.5. Anti-Glycemic, Anti-Hypertensive, and Anti-Inflammatory Assays

#### 2.5.1. Dipeptidyl Peptidase-IV (DPP-IV) Inhibition

DPP-IV inhibiting activity was determined, following the method described by Lacroix and Li-Chan [16], with some modifications [7]. Samples were reconstituted in 100 mM Tris-HCl buffer (pH 8.0). In a 96-well plate, 25 μL of the sample solution was pre-incubated with 25 μL of substrate Gly-Pro p-nitroanilide hydrochloride (6 mM), at 37 °C for 10 min. To initiate the reaction, 50 μL of human DPP-IV (4.5 unit/mL) was added and incubated at 37 °C for 1 h. The absorbance of liberated p-nitroanilide was measured at 405 nm. Ile-Pro-Ile, DPP-IV inhibitor, was used as a reference, following the same procedure. The positive control used a buffer in place of the inhibitor, while the negative control used a buffer in place of DPP-IV.

#### 2.5.2. Alpha-Amylase Inhibition

The inhibition of α-amylase activity was assayed using soluble starch as a substrate, according to the modified procedure by Awosika and Aluko [17]. Samples were dissolved in 1 mL of 20 mM sodium phosphate buffer (pH 6.9), containing 6 mM NaCl. In brief, 100 μL aliquot of each sample and 100 μL of α-amylase solution (1 mg/mL) were added to the test tubes and allowed to incubate for 10 min at 25 °C. After incubation, 100 μL of a 1 g/100 mL starch solution (dissolved in the above buffer) was added and incubated at 25 °C, for another 10 min. The reaction was terminated by adding 200 μL of dinitrosalicylic acid (DNSA) color reagent (96 mM DNSA, 2 M sodium potassium tartrate tetrahydrate and 2 M NaOH), followed by incubation in a boiling water bath at 100 °C, for 5 min. Reactions were diluted with double distilled water (3 mL), then the enzyme activity was quantified by measuring the maltose equivalents released from starch at 540 nm. A blank reading (buffer in place of enzyme) was subtracted from each absorbance. A pharmacological α-amylase inhibitor (acarbose) was assayed using the same protocol and used as a positive control.

#### 2.5.3. Alpha-Glucosidase Inhibition

Inhibition of α-glucosidase was assayed according to the methods previously described by Shobana et al. [18], with modifications [17]. The enzyme solution was prepared by homogenizing rat intestinal acetone powder (300 mg) in 9 mL of 0.9% (w/v) NaCl solution, centrifuged, and filtered. The reaction mixture consisted of substrate p-nitrophenyl-α-d-glucopyranoside (25 µL, 10 mM), PBS (pH 6.9, 25 µL, 0.1 M) and a sample (50 µL) preincubated at 37 °C, for 10 min, in a 96-well plate. The reaction was initiated by adding rat intestinal α-glucosidase solution (50 µL; 1.0 U/mL) and then incubated at 37 °C for 30 min. The absorbance of p-nitrophenyl release was measured at 405 nm. Acarbose was assayed with the same procedure and used as a positive control. The positive control used a buffer in place of the inhibitor, while the negative control used a buffer in place of α-glucosidase.

#### 2.5.4. Angiotensin Converting Enzyme II Inhibition 

ACE-inhibitory activity was measured using the method described by Martínez-Alvarez et al. [19], with modifications [7]. The substrate Hippuryl-His-Leu (HHL) was hydrolyzed by ACE to hippuric acid (HA) and histidyl-leucine (HL). The relative amounts of liberated HA and HHL not cleaved were measured. Reactants were dissolved in 100 mM sodium phosphate buffer (pH 8.3) with 300 mM NaCl. Samples (25 µL) were preincubated with 25 μL of substrate (HHL) for 4 min, at 37 °C, then 80 μL of ACE (5 mU) was added, incubated at 37 °C for 120 min. The reaction was terminated with 50 µL of 1 M HCl, then, the solution was filtered with 0.22 µm nylon filters. The positive control reaction contained 25 µL buffer in place of inhibitor (CPH). The amount product HA were quantified by HPLC (Model 2690, Waters Corporation, Milford, MA, USA) on an analytical C18 column XBridge™ BEH130 C18 column.

#### 2.5.5. RAW 264.7-NF-κB Cell Culture, Cell Viability, and Anti-inflammatory Response

The NF-κB luciferase reporter cell line (Novus Biologicals) was cultured in DMEM supplemented with 4 mM L-glutamine, 1 mM sodium pyruvate, 10% FBS, 100 U/mL penicillin, 100 μg/mL streptomycin, and 3 μg/mL puromycin. All cell lines were maintained at 37 °C with 5% CO_2_. Cell viability was estimated by the MTT assay standard protocol [20], with modifications. Cells (3 × 10^4^/well) were seeded in 96-well plates and treated with various sample concentrations (in phosphate buffered saline (PBS)). After incubation for 24 h, a 20-μL aliquot of the MTT solution (5 mg/mL in PBS) was added to each well and incubated for a further 4 h at 37 °C. The purple formazan was diluted with 150 μL dimethyl sulfoxide. The absorbance was measured at 490 nm, using a microplate reader. The percentage of viable cells was calculated with respect to the cells treated with phosphate-buffered saline (PBS).

The NF-κB Luciferase reporter RAW 264.7 cell line (Novus Biologicals), which expresses an optimized Renilla luciferase reporter gene (RenSP) under the transcriptional control of an NF-κB response element, was used. The cells were seeded (2 × 10^5^ cells/well) into 24-well plates for 16 h, pretreated with samples for 1 h, then stimulated with Lipopolysaccharides (LPS; 500 ng; E. coli Serotype R515, Re, TLR grade) for 6 h. Media from each well was aspirated, and then 100 μL PBS was added to each well. Luciferase assays were performed using the Promega Renilla Luciferase detection assay kit, following the manufactures’ protocol. Luminescence was measured as the relative luminescence units (RLU) using Spectramax (Molecular Devices, San Jose, CA, USA). Control cells were treated with the media alone. 

### 2.6. Identification of Cationic Peptides and Molecular Docking

Sample preparation, mass spectrometry analysis, bioinformatics, and data evaluation were performed in collaboration with the Proteomics Core Facility at the Indiana University School of Medicine. Methods are described in literature reports published elsewhere [21,22] and vendor provided protocols. Samples were analyzed using a 5 cm trap column and a 15 cm (2 µm particle size, 50 µm diameter) EasySpray (801A) column on an UltiMate 3000 HPLC and Q-Exactive Plus ((ThermoFisher Scientific, Walthman, MA, USA) mass spectrometer. Data analysis, including de novo, were performed using the PEAKS software (Bioinformatics Solutions), with Q-Exactive. From the generated peptides, only peptide with an Average Local Confidence (ALC) >80% were chosen to proceed to the following step. The peptide list was searched against the BIOPEP-UWM Database of Bioactive Peptides [23]. Antihypertensive (AHT) prediction and amphiphilic scores were generated by the AHTPin [24] in silico prediction algorithm of antihypertensive peptides (Supplementary Table S1). Molecular docking was performed using the Accelrys Discovery Studio software and Auto Dock Vina, as previously described [25]. Among all identified peptides, all were predicted as antihypertensive peptides and were docked against ACE, along with the inhibitor drug captopril. For molecular docking, the crystal structure of human ACE bound with Lisinopril (PDB: 108A) was used. Before docking, Lisinopril and water molecules were removed, and polar hydrogen atoms were added in the presence of cofactors (zinc and chloride ions). A binding site with a radius of 15 A and coordinates x: 41.58, y: 37.374, and z: 43.47 was created. All generated docking modes were evaluated according to the affinity energy values.

### 2.7. Statistical Analysis 

All data are presented as mean ± SD from 3 to 8 independent experiments, unless otherwise indicated. Data analyses were performed by one-way analysis of variance (ANOVA), coupled with Tukey’s post-hoc test, using the PRISM 6 statistical software (Graph Pad Software, San Diego, CA, USA). *p* < 0.05 was considered significant.

## 3. Results

### 3.1. Molecular Weight Distribution of CPH and CPHD

**Figure 1:**
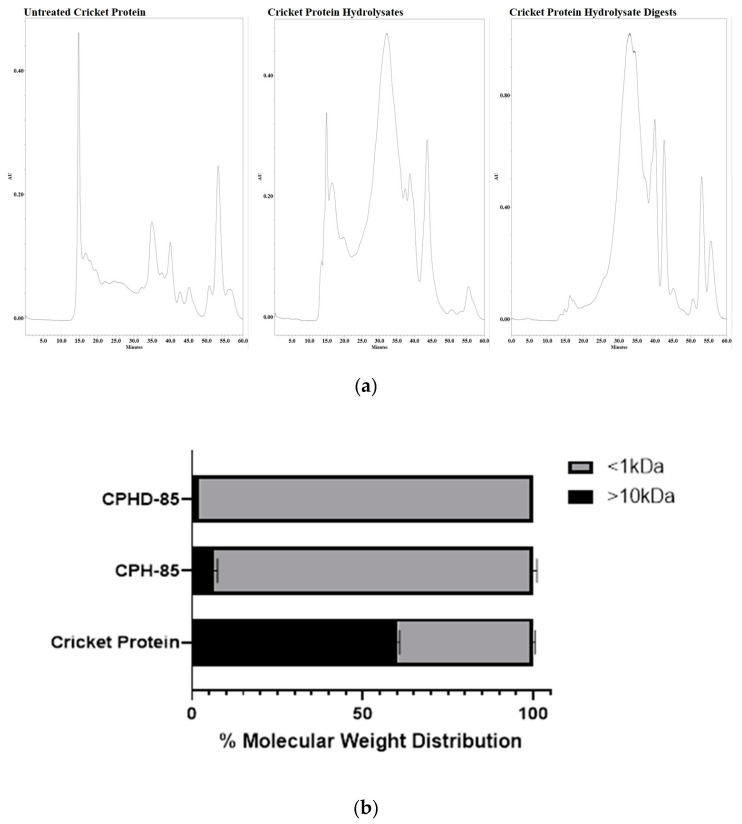
Molecular weight distribution of cricket protein after enzymatic hydrolysis and in vitro digestion. (**a**) Gel filtration peptide profile of non-hydrolyzed cricket protein, cricket protein hydrolyzed with alcalase, and cricket protein hydrolysates (CPH), after digestion with gastrointestinal enzymes. (**b**) Gel filtration peptide profile of non-hydrolyzed cricket protein, cricket protein hydrolyzed with alcalase, and CPH, after digestion with gastrointestinal enzymes.

After controlled proteolysis with the commercial enzyme alcalase, the cricket protein hydrolysate (CPH) was subjected to in vitro simulated gastrointestinal digestion (SGID). Alcalase proteolysis, followed by SGID, generated a complex mixture of peptide-rich digests (CPHD). Before enzyme treatment, more than 50% of the native cricket proteins were above 10 kDa (Figure 1b) The alcalase proteolysis (3.0% E:S; 80 min) resulted in CPH consisting mostly of peptides <1 kDa (Figure 1a). After SGID, the composition of <1 kDa was more than 90%. The size exclusion chromatograms (Figure 1b) visually illustrate the changes in molecular weight distribution of the untreated cricket protein, cricket protein hydrolysates (CPH), and its digests (CPHD), after SGID. The untreated cricket protein chromatogram exhibited more peaks at earlier retention times (10–25 min), as compared to the hydrolysate mixtures. Both CPH and CPHD chromatograms showed an absence of the peaks seen in cricket protein, and instead showed the presence of a larger cluster of peaks at a later retention time (25–55 min), indicating substantial protein hydrolysis and generation of smaller peptides. The peptide molecular weight distribution of CPH and CPHD was mostly similar, indicating some resistance to digestion by the gastrointestinal proteases used in the SGID.

### 3.2. CPHD Peptide Fractionation

In our preliminary observations, the simulated gastrointestinal digests (CPHD) demonstrated an inhibition capacity to the enzymes involved in disease pathways, such as hypertension, type-2 diabetes, and inflammation [3]. Hence, the CPHD was separated by sequential chromatography to isolate and characterize the peptide or group of peptides responsible for the observed bioactivity. First, peptides were distinguished by size on a gel filtration Superdex^®^ Peptide 10/300 column (Supplementary Materials Figure S2a). Four peak fractions were collected (S1–S4), lyophilized, then probed against DPP-IV, ACE, and α-glucosidase for screening (Table 1).

Taken together, fraction S4 illustrated superior inhibition activity against all enzymes (Table 1). Next, fraction S4 was separated by reverse phase chromatography (C18), resulting in six fractions, F1 to F6. Of these fractions, F6 showed better inhibiting activity towards the three enzymes, as it was the fraction with the most hydrophobic peptides present within the CPHD (Table 1). Next, the fraction F6 was concentrated for a final separation via ion-exchange, which resulted in two peaks belonging to either anionic or cationic peptides (Supplementary Materials Figure S2c).

### 3.3. Cationic Peptide Fractions Shows Multifunctional Bioactivity

To assess the extent of its multifunctional ability, the cationic fraction was tested against ACE, DPP-IV, α-amylase, and α-glucosidase (Table 2). The DPP-IV inhibiting capacity was greater than 30% for all concentrations tested, never more than 50%, yet still exceeded the activity of the anionic fraction. The superior inhibiting activity was also observed for α-amylase, α-glucosidase, and ACE, with IC_50_ values of 18.5, 13.9, and 1.9 µg/mL, respectively. In contrast, the anionic fraction IC_50_ values were 5082.7 µg/mL (α-amylase), 76.62 µg/mL (α-glucosidase), and 509 µg/mL (ACE), indicating that the most potent peptides were present in the cationic fraction (Table 2). The data also illustrated that bioactive efficacy increased as fractionation progressed. Peptide fractions displayed enhanced inhibiting ability after being separated into charged groups (anion and cation), as compared to peptide fractions after SEC and C18 separation. This was true for all enzymes tested, except DPP-IV.

#### NF-κB Expression in Macrophage (RAW 264.7) Cells

**Figure 2:**
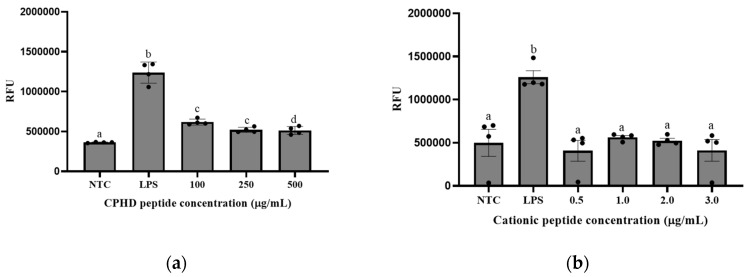
Peptides from (**a**) CPHD and (**b**) its cationic peptide fraction inhibits LPS-induced inflammation in RAW 264.7 Cells. Different lowercase letters indicate significant differences between treatments (*p* < 0.05). Samples were analyzed in quadruplicate (n = 4). Cells were stimulated for 6 h with 500 ng of lipopolysaccharide (LPS) or pre-treated with CPHD (**a**), cationic fractions, or PBS (NTC = non-treated control). The data are expressed as the Renilla luciferase activity in relative light units (RLU). Data represent the mean ± SD of quadruplicate samples, representative of four independent experiments.

**Figure 3:**
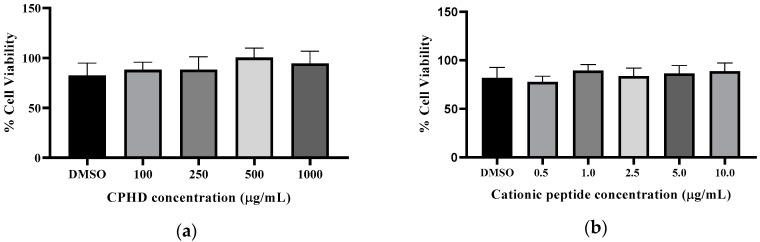
Percentage of macrophage cell viability after exposure to (**a**) CPHD and (**b**) its cationic peptide fraction. CPHD = cricket protein hydrolysates after simulated digestion with gastrointestinal enzymes. Cells were incubated with CPHD (100–1000µg/mL), cationic peptide fraction (0.5–10 µg/mL), or DMSO (0.25%), for 12 h, to evaluate cytotoxicity by MTT assay, with peptides. There was no significant (*p* > 0.05) reduction in viability, as compared to the DMSO-treated cells. Data represent the mean ± SD representative of eight (n = 8) independent experiments.

Treating RAW264.7 macrophage cells with LPS resulted in a significant increase (*p* < 0.05) in inflammatory induction, compared to that in the non-treated control. Treatment of RAW264.7 macrophage cells with the cationic fraction at various concentrations resulted in a non-concentration-dependent inhibition of LPS-induced activation (Figure 2). There were significant differences (*p* < 0.05) between the positive control (LPS only) and those dosed with CPHD and LPS. However, this inhibiting activity did not prove to be dose-dependent, showing minor variation after treatment with 0.5, 1, 2, or 3 µg/mL of the cationic peptide fraction. Comparing the inflammatory response between CPHD and its cationic fractions, CPHD also inhibited NF-κB (Figure 2a) in the presence of LPS activation. The inhibitory trend remained, but required a much higher concentration of CPHD (100–500 µg/mL) than cationic peptides (0.5–3 µg/mL). Notably, the NF-κB defect observed by CPHD and the cationic fractions were not due to the cytotoxic effects (Figure 3). Damage to the macrophages did not appear to be significant up to 10 µg/mL of cationic peptides (Figure 3a) and 1 mg/mL of the hydrolysate digests (CPHD), after 12 h exposure (Figure 3b).

### 3.4. Peptide Identification and Molecular Docking against ACE

**Figure 4:**
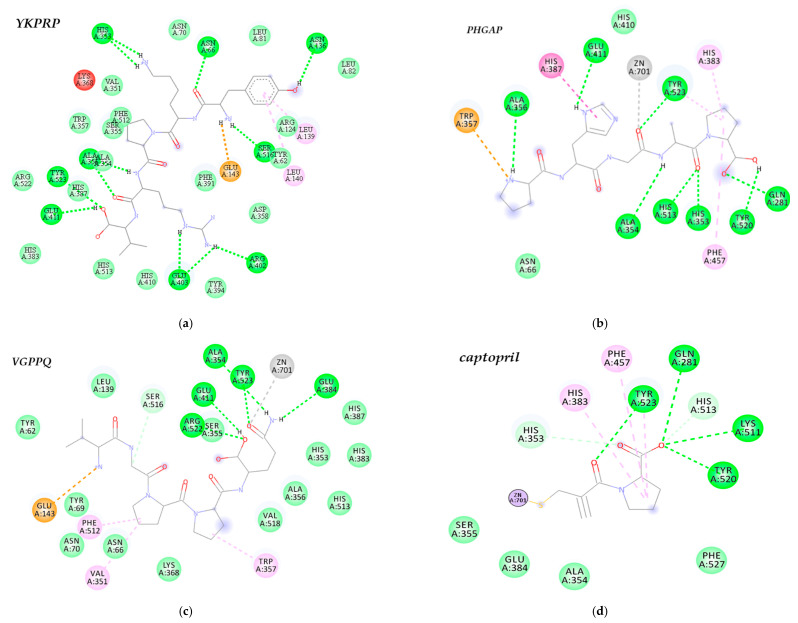
2D model of the predicted binding mode of (**a**) YKPRP, (**b**) PHGAP, (**c**) VGPPQ, and (**d**) captopril to the angiotensin-converting enzyme (ACE). Images were obtained with Discovery Studios Visualizer Software and the best scored docking pose is shown.

Twenty-eight peptide sequences were identified within the cationic peptide fraction between 384.52 and 659.84 Da and 4–6 amino acids (Supplementary Materials Table S1). A search against the BIOPEP database revealed that most of the peptides contained known ACE-inhibiting tri-peptides within their sequence. Other bioactive properties were also identified, including antioxidant, DPP-IV inhibition, and immunomodulation. Since all peptides were predicted to possess antihypertensive (AHT) capabilities [24], these were used in molecular docking studies against human ACE and compared to known ACE inhibitor captopril. A total of three peptides YKPRP, PHGAP, and VGPPQ had the lowest binding energy when bound to ACE and were mainly stabilized by hydrogen bonds and van der Waals interactions with ACE residues (Supplementary Materials Table S2). Peptide YKPRP (−39.75 kJ/mol) had a lower binding energy compared to PHGAP (−36.82 kJ/mol), VGPPQ (−36.87.26 kJ/mol), and captopril (−24.26 kJ/mol). The ACE molecule includes three main active sites; S1 with three residues Ala354, Glu384, and Tyr523; S2 comprising Gln281, His353, Lys511, and His513; and an S1’ pocket including residue Glu162 [26]. YKPRP formed multiple H-bond (Figure 4a), some of which were interactions with S1 (Tyr523, and Ala354) and S2 (His513) active site residues. Although there were not as many hydrogen bond and hydrophobic interactions, PHGAP mainly interacted with Tyr523, Ala354 residues of S1 and Tyr520, His353, His513, Gln281 of S2’, and Phe457 of the hydrophobic pocket (Figure 4b). Likewise, VGPPQ showed a high degree of interactions with S1 (Ala354, Glu 384, Tyr523) and S2 (His352, His513) residues, most of which were van de Waals in nature (Supplementary Materials Table S2). The docking results also showed interactions between peptides PHGAP and VGPPQ and Zn^2+^, at the enzyme active site, with distances less than 3.0 A to the carbonyl oxygen of the peptide. These interactions were similar to that observed of captopril, having all interactions with the ACE S1 and S2 residues of the hydrophobic pocket (Phe 457, Phe527) and Zn^2+^ coordination on the sulfhydryl (Figure 4d). 

## 4. Discussion

Cardiovascular disease is the major cause of morbidity and mortality in chronic glycemia and hypertension [27,28]. As such, diabetes and hypertension are closely linked by similar risk factors, such as endothelial dysfunction, oxidative stress, arterial atherosclerosis, obesity, and inflammation processes [27]. Diabetic and hypertensive pathways also overlap some cardiovascular complications related primarily to micro- and macrovascular disease [27]. Common mechanisms, such as upregulation of the renin-angiotensin-aldosterone system, oxidative stress, inflammation, and activation of the immune system likely contribute to the close relationship between diabetes and hypertension. Nutritional interventions were consistently proposed as a part of a comprehensive strategy to lower the incidence and severity of cardiovascular diseases and their risk factors [1]. In addition to the immense benefits of phenolic and non-peptic compounds, bioactive peptides are considered a viable treatment and prevention alternative, owing to their inhibitory nature of various disease-related enzymes [2]. Edible insects are undoubtedly an underexploited source of protein and bioactive peptides. Literature evidence shows that the most therapeutically effective edible insect preparations are in the form of peptides derived from protein hydrolysates, which showed inhibitory effects towards inflammation, hypertension, diabetes, and microbial growth [9].

In our previous work, we evaluated the potential antioxidant, ACE, and DPP-IV inhibiting capabilities of various cricket protein hydrolysates (CPH), using the commercial enzyme alcalase. CPH prepared with the highest enzyme concentration (3.0%) and incubated for 80 min, showed the best bioactivity among other hydrolysate preparations. The observed activity remained or improved after SGID, suggesting further liberation of active peptides or stability towards gastrointestinal enzymes [7]. This study highlighted the conditions necessary to produce cricket protein hydrolysates that might be biologically active. However, the peptide/peptide group responsible was not yet characterized. The main objective of this study was to isolate and characterize peptide groups present within cricket protein hydrolysates, primarily responsible for its potential bioactivity. In general, active enzymatic hydrolysates contain peptides with a wide range of molecular weights, amino acid sequences, hydrophobicity and hydrophilicity, charge, and bioactive efficacies; therefore, this work focused on assay-directed fractionation. The SGID digests of the protein hydrolysates (CPHD) were chosen as the starting material for fractionation, since peptides likely exhibit their properties after digestion and absorbance into the blood system. Following the data described in Figure 1 and Table 1, the CPHD were first separated by molecular weight size. There were four major groups of peptides (Fractions S1–S4) with distinguished molecular weights and retention time. Fraction S4 was identified as having the best inhibiting activity towards ACE, DPP-IV, and α-glucosidase, and was further separated using RP–HPLC. From the chromatographic profile at 214 nm (Supplementary Materials Figure S1a), fraction S4 was still a complex mixture of peptides, which was then separated according to the hydrophobicity of the peptides (F1–F6). ACE and α-glucosidase inhibition assays revealed that fraction F6 had better inhibition activity compared to the other fractions. After two stages of fractionation, S6 and F4 likely contained hydrophobic peptides below 10 amino acids, similar to known biologically relevant peptides [2]. Food-derived bioactive peptides usually do not have any specific consensus on amino acid sequences that are responsible for their biological activity, but most maintain certain common features, such as having a positive/neutral charge and relatively hydrophobic and amphipathic structure [29]. Likewise, smaller molecular weight peptides (2–10 residues) are more soluble and can coordinate efficiently with enzyme active site regions [2]. This trend corresponds well with that described by Vercruysse et al. [6], where peptide fractions from cotton leaf worm (*S. littoralis*) were separated by SEC and RP–HPLC. Fractionation revealed an active peptide with an aromatic, hydrophobic amino acid at the carboxyl terminus, namely phenylalanine, and consisted of a hydrophobic amino acid at the amino terminus, alanine, which showed antihypertensive activity in vitro and in vivo [6]. Fraction F6 was separated into two charged groups, anionic and cationic peptides, which confirmed that the most potent peptides were primarily cationic in nature. The data conferred the cationic pool of peptides with superior inhibiting activity towards ACE, α-amylase, and α-glucosidase; DPP-IV inhibiting activity, however, decreased after the various stages of fractionation.

Cellular inflammation and dysfunction play a crucial role in several disorders, including CVD risk factors, such as hypertensive and hyperglycemic responses. Nuclear factor-kappa-light-chain-enhancer of activated B cells (NF-κB) is a central regulator of pro-inflammatory mediators and is an essential component linking chronic inflammation and CVD. Thus, regulating NF-κB is considered a potential therapeutic target for CVD risk factors [30]. Since the monocytogenes-induced NF-κB pathway is the most dominant host response in macrophage cells, we examined the ability of cricket peptides to inhibit NF-κB in the RAW 264.7-NF-κB-luciferase reporter cell line. Our results demonstrated that the cationic peptide fraction inhibited NF-κB expression in RAW 267.4 cells. This reporter contained a firefly luciferase gene under the control of multimerized NF-κB responsive pathways, which are commonly used to assess the potency of anti-inflammatory molecules and peptides. During inflammatory scenarios, NF-κB positively modulates expression levels of several chemokines (e.g., MCP-1), proangiogenic factors, for example, vascular endothelial growth factor (VEGF), and cellular inhibitors of apoptosis [31]. In this study, NF-κB expression was stimulated by the bacterial cell wall component lipopolysaccharide (LPS), which is known to induce an inflammatory cascade, by activating the TLR4-NFκB signaling pathway [30]. Cells pre-treated with the cationic fraction demonstrated lower levels of NF-κB, as compared to LPS alone, verifying that this fraction contained some of the more potent peptides present within CPHD. This observation was further confirmed by the fact that the isolated cationic fractions exhibited proportionate NF-κB regulation, compared to that of CPHD (Figure 2). Notably, this anti-inflammatory activity was maintained and required a much lower concentration of the cationic peptides (1–10 µg/mL vs. 100–500 µg/mL, Figure 2). Most food-derived peptides exert anti-inflammatory activities by inhibiting signaling components of either NF-κB or mitogen-activated protein kinase (MAPK) pathways, the two major pathways were involved in chronic inflammation following uncontrolled signal activation [32]. Similar to the findings of this study, venom from wasp (*Nasonia vitripennis*) dose-dependently inhibited NF-κB signaling in reporter fibrosarcoma cells [33]. More than half of NF-κB was suppressed with 6.4 and 12.5 µg/mL of wasp venom. In addition to the NF-κB inhibitory action, the wasp venom also inhibited IL-6 (a pro-inflammatory cytokine) expression in LPS-activated macrophages, as well as, expression of two NF-κB target genes, IκBα and A20 [33]. In another study, peptide fractions from cricket (*G. sigilattus*) protein hydrolysates also demonstrated anti-inflammatory properties [34]. The study detailed the efficacy of SEC isolated peptide fractions to inhibit lipoxygenase and cyclooxygenase-2 activity with IC_50_ values of 0.13 µg/mL and 0.26 µg/mL, respectively. The authors also noted that better anti-inflammatory activity was observed for the peptide fractions, as compared to the hydrolysates [34]. 

Cationic bioactive peptides, particularly, were shown to be useful therapeutic agents against human diseases, when exhibiting high bioactive potency and no cytotoxic activity against mammalian cells. Structure-function analyses revealed that the amphipathic structure, positive charge, and hydrophobic properties help facilitate their interaction with cell walls and inserting past the membrane to the site of action [35]. The de novo sequencing revealed that the cationic peptides fractioned from CPHD consisted of peptides with these characteristics and were predicted as hypotensive peptides (Supplementary Materials Table S1), which might have played a role in the superior ACE inhibiting activity exhibited by this fraction (Table 3). Additionally, most identified sequences contain tri-peptides with reported activity, including antioxidant, antimicrobial, anti-glycemic, and immunomodulatory properties [23]. This would explain the multifunctional ability of the cationic peptide fraction observed (i.e., ACE, α-glucosidase, α-amylase, and NF-κB inhibition) in this study. Among the peptides identified, the molecular docking results revealed that the pentapeptides YKPRP, PHGAP, and VGPPQ had binding properties similar to those of commercial ACE, inhibiting drug captopril with different potentialities (Table 3). All three peptides had binding energies lower than captopril, but similar to that of Lisinopril (−39.96 kJ/mol) [36]. 

The ability of peptides to form multiple hydrogen bonds and hydrophobic interactions is a key characteristic of peptide-induced inhibition, through stabilization of the non-catalytic enzyme-peptide complex structure [37]. All three peptides interacted with ACE active sites mainly via hydrogen bonding and van der Waals interactions. However, peptides PHGAP and VGPPQ showed more interaction with S1 and S2 active site residues. In addition to hydrogen and hydrophobic bonds, the interaction between the Zn^2+^ ion and peptides plays a significant role in ACE inhibition [37]. Both pentapeptides PHGAP and VGPPQ also exhibited interaction with ZN^2+^ atoms, binding to ACE in an analogous fashion to that observed in captopril (Figure 4), indicating that they would be potent ACE inhibitors. Nevertheless, YKPR is a known neuropeptide, an analogue of the natural immunomodulatory tetrapeptide tufsin (TKPR), and might be active towards hormonal and neurological functions. Similar peptides are reported as neuromodulators isolated from various insect species and other crustaceans, such as lobster and crabs [38]. For example, the penta-peptide RYLPT (proctolin) is the first insect neuropeptide sequenced and chemically characterized. However, a study demonstrated that proctolin, although showed a high degree of binding affinity towards ACE, was resistant to hydrolysis in vivo. [39]. Peptides, like proctolin, which have a prolyl residue near the C-terminal are known to be resistant to hydrolysis by mammalian ACE, due to the structural constraints imposed by the presence of imino acids [39]. This is likely why YKPRP, in this study, demonstrated a lower binding affinity than other peptides, yet less interaction with active site residues. Whereas peptides PHGAP and VGPPQ contained tripeptides GAP and GPP, respectively. These tripeptides are present in many ACE-inhibiting, antioxidant, DPP-IV-inhibiting, and antimicrobial food-derived peptide sequences (Supplementary Materials Table S1) [23]. Additionally, potent ACE inhibitors include hydrophobic amino acid residues near the C-terminal. Proline is reported as a desirable residue, but also aliphatic peptides such lysine is responsible for the ACE-inhibitory capacity. The molecular docking interactions in this study also corroborate kinetic analyses in our previous work, where cricket peptides demonstrated mixed-type inhibition towards ACE, indicating that binding was possible with both free enzyme and enzyme-substrate complex, to suppress ACE activity [40]. YKPRP, PHGAP, and VGPPQ all coordinate with residues within the active site, as well as non-catalytic residues. This suggests that these peptides might be able to stabilize in an inactive state, even if bound to its target substrate. Zielińska et al. [41] also isolated ACE inhibitory peptides from cricket (*G. sigillatus*) protein hydrolysates. They identified the sequences IIAPPER, LAPSTIK, KAPEEHPV, KVEGDLK, which showed no homology to the peptides identified in this study. These differences were attributed to conditions used to liberate bioactive peptides and pretreatment techniques, also demonstrated in their study (i.e., raw vs boiling before extraction). To the best of our knowledge, the bioactive pentapeptide sequences derived from this study using CPHD were not previously reported. Based on the results obtained in this study, analyzing the molecular mechanism involved between these identified peptide sequences on hypertensive, glycemic, and inflammatory responses, as well as their in vivo bioactive effects, will be the focus of future studies. Considering the variety of activity displayed in this study, enhanced potency post-fractionation, and affinity of novel peptides towards ACE (a central regulator of the renin–angiotensin aldosterone system and known target for treating hypertension) highlights that cricket-derived peptides could play a significant beneficial role in the pathology involved in cardiovascular dysfunction.

## 5. Conclusions

In this study, we selected cricket protein hydrolysates as a starting material to characterize and identify potent bioactive peptides upon simulated gastrointestinal digestion. After separation by size and hydrophobicity, fractions were screened for enzyme inhibitory capacity, then finally separated by charge. Subsequently, we examined the antihypertensive, antidiabetic activity, and anti-inflammatory activity of the final fraction and identified the peptide sequences, using LC–MS/MS. 

Taken together, cricket protein hydrolysates contain potent peptides with potential ACE, α-glucosidase, and α-amylase inhibiting capacity. Additionally, CPHD and its cationic fractions inhibited LPD-induced NFκB activity, but other molecular events such as peptide-TLR4 interaction remain unknown. Despite the effect of the protein hydrolysates and cationic peptide fractions on the NF-κB expression in macrophage cells, it should be noted that we did not observe a clear relationship between sample concentration and their biological effect. Notably, the sequences of ACE-inhibitory peptides were identified as three pentapeptides, YKPRP, PHGAP, and VGPPQ. Consumption of edible cricket peptides, alone or in the form of functional foods, might contribute to positive effects towards conditions associated with inflammation and hypertension. Nevertheless, these results are a contribution to future research, which should be undertaken to verify the findings, using in vivo models and clinical trials. Moreover, these results could be the basis for further research on other species of edible insects.

## Figures and Tables

**Table 1:** Percentage inhibition of angiotensin converting enzyme (ACE), dipeptidyl peptidase-IV (DPP-IV), and α-glucosidase by CPH-digests (CPHD) peptide fractions.

	Fraction	ACE (%)	DPP-IV (%)	α-Glucosidase (%)
**Size exclusion**	S1	32.3 ± 0.31 ^a^	19.3 ± 2.52 ^a^	62.2 ± 1.09 ^a^
	S2	28.8 ± 0.02 ^b^	16.98 ± 0.30 ^a^	25.9 ± 2.54 ^b^
	S3	47.5 ± 0.87 ^c^	20.6 ± 0.61 ^a^	83.6 ± 1.14 ^c^
	S4	73.9 ± 1.12 ^d^	68.0 ± 2.52 ^b^	89.5 ± 0.91 ^d^
**Reverse-phase**	F1	25.4 ± 0.6 ^e^	5.1 ± 0.01 ^c^	9.4 ± 0.36 ^e^
	F2	29.4 ± 0.04 ^g^	15.3 ± 2.70 ^d^	11.6 ± 0.04 ^f^
	F3	67.2 ± 0.12 ^h^	18.4 ± 0.59 ^a^	8.4 ± 0.47 ^g^
	F4	32.2 ± 0.19 ^a^	17.1 ± 1.61 ^a^	7.3 ± 1.51 ^h^
	F5	47.4 ± 0.5 ^c^	21.0 ± 1.96 ^a^	22.3 ± 2.79 ^i^
	F6	93.4 ± 0.08 ^i^	26.98 ± 1.63 ^e^	48.2 ± 5.79 ^j^
**Positive inhibitor**	Captopril	98.0 ± 0.01 ^j^	n.a	n.a
	Ile-Leu-Pro	n.a	88.9 ±6.05 ^f^	n.a
	Acarbose	n.a	n.a	70.9 ± 0.43 ^k^

Results are reported as the percentage of inhibiting activity and is expressed as a mean of least triplicate (n = 3) determinations, with standard deviation. S1–S4 are fractions collected from the gel filtration column. F1–F6 are fractions collected from the reverse phase C18 column. Captopril, peptide Ile-Leu-Pro, and Acarbose are commercially available therapeutic options to inhibit the angiotensin-converting enzyme II, Dipeptidyl peptidase-4, and α-glucosidase, respectively. n.a. = not applicable. CPHD = CPH after simulated digestion with gastrointestinal enzymes. Lowercase letters indicate significant differences between fractions within assays (*p* < 0.05).

**Table 2:** IC_50_ values for α-amylase, α-glucosidase, and ACE inhibition by the IEX fractions of CPHD peptide fractions.

IEX Fraction	α-Amylase (µg/mL)	α-Glucosidase (µg/mL)	ACE (µg/mL)
Cationic Peptides	18.537	13.902	1.922
Anionic Peptides	5082.75	76.623	509.062

IC_50:_ The peptide concentration required to inhibit 50% of enzyme activity. CPHD = cricket protein hydrolysates after simulated digestion with gastrointestinal enzymes.

**Table 3:** Predicted binding energies, ZN(II) coordination, and ACE residues that interact with the docked ligands.

Ligand	Affinity Energy (kJ/mol)	Zn (II) Coordination	Interaction with ACE Active Site Residues *							
			Glu384	Tyr523	Ala354	Gln281	Tyr520	Lys511	His513	His353
Captopril	−24.26	sulfhydryl group of captopril	+	+	+	+	+	+	+	+
YKPRP	−39.75	None	−	+	+	−	−	−	+	+
PHGAP	−36.82	carboxylic acid group of glycine	−	+	+	+	+	−	+	+
VGPPQ	−36.87	carboxylic acid group of lysine	+	+	+	−	−	−	+	+

* Interactions are denoted with a “+” indicating that there was an interaction between the ACE residue and ligand or “−“indicating no interaction.

## Supplementary Materials

The following are available online at https://www.mdpi.com/2072-6643/12/11/3588/s1. Figure S1: Representative elution profiles illustrating fractions collected from gel filtration (SEC) (a), reverse phase (C18) (b), and semi-preparative cation exchange (IEX) (c) columns. Table S1: De novo peptide list, BioPEP database matches, and antihypertensive peptide prediction. Table S2: All residues interacting with ACE (PDB 108A) for each peptides YKPRP, PHGAP, VGPPQ, and known ACE inhibitor captopril. Table S3. Predicted binding affinity (kJ/mol) for best-docked pose of peptides, against ACE.
