# Supplementary material for: Identification and Characterization of Edible Cricket Peptides on Hypertensive and Glycemic In Vitro Inhibition and Their Anti-Inflammatory Activity on RAW 264.7 Macrophage Cells
Source: Nutrients. 2020 Nov 23;12(11):3588. doi: 10.3390/nu12113588 (PMC7700588; doi:10.3390/nu12113588)
Supplement: Supplementary file 1 [file nutrients-12-03588-s001.pdf]

Supplementary Materials for

Identification and Characterization of Edible Cricket Peptides on Hypertensive and Glycemic *in vitro* Inhibition and Their Anti-Inflammatory Activity on RAW 264.7 Macrophage Cells

Felicia Hall[1,2], Lavanya Reddivari[1], and Andrea M. Liceaga[1,2]*
[1]Protein Chemistry and Bioactive Peptides Laboratory; Purdue University, West Lafayette, IN, 47907, USA;
[2]Department of Food Science, Purdue University, 745 Agriculture Drive, West Lafayette, IN, 47907, USA; lreddiva@purdue.edu

Contact information for Corresponding Author
*Andrea M. Liceaga, Email: aliceaga@purdue.edu, Tel. 765-496-2460

Supplementary Materials for this manuscript include the following:

Figure S1: Representative elution profiles illustrating fractions collected from gel filtration (SEC) **(a)**, reverse phase (C18) **(b),** and semi-preparative cation exchange (IEX) **(c)** columns. Chromatography was performed sequentially as follow: CPHD was separated by gel filtration (a); fraction S4 collected and separated by reverse-phase chromatography (b); and fraction F6 collected and separated by IEX. Elution profiled monitored at 280nm.

Table S1: De novo peptide list, BioPEP database matches, and antihypertensive peptide prediction. Characterization parameters include de novo score, average local confidence (%) peptide abundance (area). Database searches were performed using PEAKS software. Antihypertensive peptide predication includes SVM score, prediction (AHT or non-AHT), and amphiphilic score. Peptides searched against the BioPEP database reports the known peptide and bioactivity reported.

Table S2: All residues interacting with ACE (PDB 108A) for each peptides YKPRP, PHGAP, VGPPQ and known ACE inhibitor captopril.
Table S3. Predicted binding affinity (kJ/mol) for peptides best docked pose against ACE

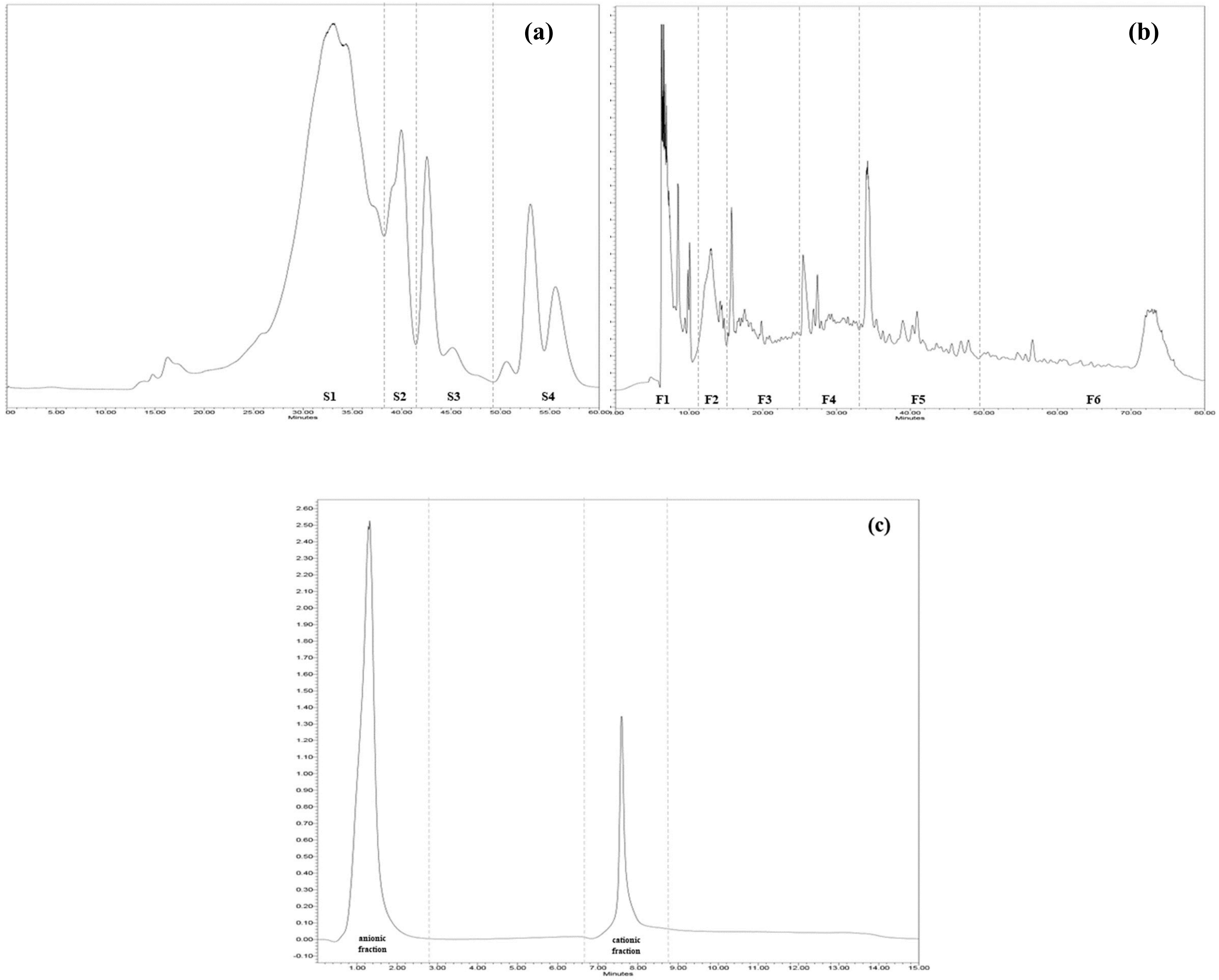
(a)
S1
S2
S3
S4
Minutes
(b)
F1
F2
F3
F4
F5
F6
Minutes
(c)
anionic fraction
cationic fraction
Minutes

Table S1. De novo peptide list, BioPEP database matches, and antihypertensive peptide prediction

Peptide Sequence	De Novo Score	ALC (%)	Area	SVM Score	Prediction	Amphiphilicity Score	Mol wt.	Matched Peptide in BioPEP Database	Activity Reported
**LPPK**	85	85	1.76E+06	2.25	AHT	0.92	453.62	PPK	ACE inhibitor
**GPPK**	81	81	3.91E+06	2.05	AHT	0.92	397.52	GPP, PPK	ACE inhibitor
**YKPRP**	95	95	1.58E+06	2.02	AHT	2.24	659.84	exact match	immunomodulating
**VLPQ**	85	85	1.70E+07	1.59	AHT	0.31	455.6	VLP	ACE inhibitor
**VKLAPG**	83	83	2.01E+07	1.54	AHT	0.61	583.8	VKL, APG, LAP	DPPIV and ACE inhibitor
**LLPQ**	85	85	5.00E+06	1.43	AHT	0.31	469.62	LLP	ACE inhibitor
**LQPL**	89	89	4.38E+06	1.43	AHT	0.31	469.62	LQP	ACE and DPP-IV inhibitor
**PHGAP**	80	80	9.73E+05	1.35	AHT	0.29	477.58	PHG	not specified
**VGPPQ**	92	92	1.00E+07	1.33	AHT	0.25	496.63	GPP	ACE inhibition, antioxidant
**FVPL**	87	87	4.10E+06	1.29	AHT	0	474.64	FVP, VPL	ACE and DPPIV inhibitor
**VHLP**	87	87	1.69E+06	1.27	AHT	0.36	464.61	exact match	PEP inhibitor
**PLVR**	85	85	3.66E+06	1.27	AHT	0.61	483.65	LVR	ACE inhibitor
**LVRP**	85	85	3.16E+07	1.27	AHT	0.61	483.65	VRP, LVR	ACE inhibitor
**VHLP**	87	87	1.69E+06	1.27	AHT	0.36	464.61	exact match	PEP inhibitor
**FVPH**	82	82	1.07E+07	1.23	AHT	0.36	498.63	FVPH, FVP	antioxidant and ACE inhibitor
**LVPK**	83	83	2.54E+07	1.15	AHT	0.92	455.64	VPK	ACE
**LLPR**	98	98	2.25E+07	1.14	AHT	0.61	497.67	LLP	ACE
**LPLR**	95	95	2.46E+07	1.14	AHT	0.61	497.67	LPL	DPPIV inhibitor
**LRPL**	96	96	6.88E+07	1.14	AHT	0.61	497.67	LRP	ACE inhibitor
**LLPF**	80	80	1.14E+07	1.12	AHT	0	488.66	LLPF, LLP	antioxidant and ACE inhibitor
**VGPL**	84	84	2.72E+07	1.1	AHT	0	384.52	GPL	ACE inhibitor

**FVPK**	93	93	1.34E+06	1.1	AHT	0.92	489.66	VPK, FVP	ACE inhibitor
**AVPL**	81	81	3.49E+06	1.04	AHT	0	398.54	AVP, VPL	ACE inhibitor and diproin B
**VAPL**	88	88	1.56E+06	1.04	AHT	0	398.54	VAP	ACE inhibitor
**GPWL**	97	97	4.60E+05	1	AHT	1.73	471.61	PWL	synthetic peptide, activity not specified
**LLAP**	82	82	2.71E+06	0.9	AHT	0	412.56	LAP	DPPIV inhibitor
**QLGPL**	83	83	3.24E+07	0.8	AHT	0.25	526.69	LGP	not specified

Table S2. All residues interacting with ACE (PDB 108A) for each peptides YKPRP, PHGAP, VGPPQ and known ACE inhibitor captopril.

Interaction Type	Captopril	YKPRP	PHGAP	VGPPQ
**H-Bond**	Gln281	Asn66	Glu411	Ala354
	Tyr523	Asn136	Ala356	Glu384
	Lys511	His353 (2)	Tyr523	Arg522
	Tyr520	Ala354	Gln281	Ser516
	His353	Ala356	Tyr520	Glu411
	His513	Glu411	His353	
		Glu403 (2)	His513	
		Arg402	Ala354	
		Ser516		
		Tyr523		
**Vdw**	Ser355	Asn70	His410	Tyr69
	Glu348	Leu81	Asn66	His353
	Ala354	Leu80		His383
	Phe527	Arg124		His513
		Tyr62		Val518
		Phe391		Ala356
		Asp358		Lys368
		Tyr394		Arg124
		His410		Tyr62
		His513		Leu139
		His383		Asn70
		Arg522		Asn66
		His387		His387
**Other**	Phe457	Leu139	Trp357	Phe512
	His383	Leu140	His383	Val351
		Glu143	Phe457	Trp357

Table S3. Predicted binding affinity (kJ/mol) for peptides best docked pose against ACE

Peptide Sequence	Binding Affinity (kJ/mol)
**LPPK**	-31.7984
**GPPK**	-31.7984
**YKPRP***	**-39.748**
**VLPQ**	-32.6352
**VKLAPG**	-35.1456
**LLPQ**	-33.0536
**LQPL**	-37.2376
**PHGAP***	**-37.656**
**VGPPQ***	**-35.564**
**FVPL**	-33.0536
**VHLP**	-36.4008
**PLVR**	-35.9824
**LVRP**	-36.8192
**VHLP**	-37.2376
**FVPH**	-31.7984
**LVPK**	-35.564
**LLPR**	-33.472
**LPLR**	33.8904
**LRPL**	35.1456

*Peptides with the most favorable bonding interactions with ACE active site regions and/or with Zn (II) ions
